# Small molecule modulation of the p75 neurotrophin receptor promotes dendritic spine resilience to pathogenic tau species and reduces their accumulation

**DOI:** 10.1186/s40478-026-02263-5

**Published:** 2026-03-05

**Authors:** Tao Yang, Yeonglong Ay, Sukhneet Kaur, Robert R. Butler, Kevin C. Tran, Harry Liu, Vanessa F. Langness, Stephen M. Massa, Frank M. Longo

**Affiliations:** 1 Department of Neurology and Neurological Sciences, School of Medicine, Stanford University, 453 Quarry Road, Stanford, CA 94304 USA; 2 Department of Neurology, San Francisco Veterans Affairs Health Care System, San Francisco, CA 94121 USA; 3 Department of Neurology, University of California, San Francisco, San Francisco, CA 94121 USA; 4 Wu Tsai Neuroscience Institute, Stanford University, Stanford, CA 94305 USA

**Keywords:** Tau, Tauopathy, Synapse loss, Tau oligomer, Dendritic spine, p75 neurotrophin receptor (p75^NTR^), Cofilin, RhoA, LM11A-31, LIM kinase (LIMK), Slingshot phosphatase (SSH), Protein kinase C (PKC)

## Abstract

In tauopathies, such as Alzheimer’s disease (AD), oligomeric tau (oTau) can induce tau hyperphosphorylation and further oligomerization, contributing to dendrite and dendritic spine degeneration. The p75 neurotrophin receptor (p75^NTR^), which regulates signaling that overlaps with tauopathy-related degenerative pathways, is a promising therapeutic target. LM11A-31, a small-molecule p75^NTR^ modulator, downregulates p75^NTR’^s degenerative signaling and upregulates signaling that promotes dendritic and synaptic integrity. In a tauopathy mouse model, LM11A-31 reversed spine loss in hippocampal pyramidal neurons and improved performance on behavioral tests of hippocampal function. In a Phase 2a randomized, placebo-controlled AD clinical trial, LM11A-31 slowed the progression of synaptic degeneration biomarkers. In the present study, we tested the hypothesis that LM11A-31 enhances resilience of spines and dendrites to pathological forms of tau and/or inhibits the formation and accumulation of pathological tau species. We found that LM11A-31 prevented degeneration of spines and dendrites in cultured hippocampal neurons exposed to recombinant oTau or oTau-containing hippocampal extract fractions from PS19 tauopathy mice. Treating PS19 mice with LM11A-31 reduced the degeneration-promoting activity in oTau-containing hippocampal extract fractions. In cultured hippocampal neurons, LM11A-31 inhibited excess tau phosphorylation and aggregation induced by oTau and counteracted oTau-induced abnormalities in RhoA, PKC, LIMK1, and cofilin signaling, a signaling module known to regulate spine integrity. The ability of LM11A-31 to confer resilience to dendritic spines was partially dependent on LIMK1 activity. These findings demonstrate that small-molecule modulation of p75^NTR^ by LM11A-31 both reduces the accumulation of pathological tau species and promotes resilience against oTau-induced dendritic spine degeneration, exemplifying a “dual-mechanism” therapeutic approach for tauopathies.

**Supplementary Information:**

The online version contains supplementary material available at 10.1186/s40478-026-02263-5.

## Introduction

Tauopathies, such as Alzheimer’s disease (AD), are characterized by the accumulation of hyperphosphorylated tau protein species. In AD, excess phosphorylation of tau may be driven by amyloid beta (Aβ) pathology, while in primary tauopathies, pathological tau accumulation occurs independently of Aβ. Hyperphosphorylation, misfolding, and aggregation of tau protein mediate dendrite, synapse, and dendritic spine dysfunction and loss; effects on synapses and spines are closely correlated with the decline of cognitive function [15, 18, 21, 26, 27, 79]. Dendritic spines are actin-rich protrusions that form a core functional component of synapses. Strengthening or weakening of existing synapses and changes in the number and shape of dendritic spines are important mechanisms contributing to learning and memory formation [57].

Interestingly, some individuals remain cognitively healthy despite exhibiting substantial Aβ and tau pathology in the brain [97]. This cognitive resilience has been associated with preservation of synapses, particularly dendritic spines [10, 77]. Pharmacologically activating pathways that promote resilience to tau-mediated spine and synapse degeneration may represent a powerful therapeutic strategy that can be used on its own or in combination with strategies that directly reduce pathological tau species. The p75 neurotrophin receptor (p75^NTR^) represents a particularly attractive target for such a strategy.

p75^NTR^ is expressed in neuronal populations that are vulnerable in tauopathies such as AD, including those affected in the earliest stages [12, 25, 53]. Its signaling network substantially overlaps with degenerative signaling active in these diseases, positioning it as a unique lever to both promote resilience against tau pathology and reduce accumulation of pathological tau [25, 53]. p75^NTR^ can promote synaptoprotective or degenerative effects depending on the ligand, the presence of co-receptors, and the availability of intracellular adaptor proteins [14]. Binding of p75^NTR^ with mature neurotrophins can activate the phosphoinositide 3-kinase/Akt (PI3K/Akt) pathway, which promotes survival, growth, and synaptic plasticity [41, 88]. In the presence of pro-neurotrophins, which are the main p75^NTR^ ligands in the CNS, or in an unliganded state, p75^NTR^ signaling promotes activation of the pro-degenerative Jun kinase (JNK) pathway [2] or excess activation of the GTPase RhoA [43, 75, 76]. Dysregulation of RhoA-cofilin signaling occurs in AD [33, 51, 86], exacerbating tau pathology [85] and contributing to dendritic spine loss. Physiological activation of RhoA signaling results in downstream phosphorylation and inactivation of the actin-depolymerizing protein cofilin via Rho-associated kinase (ROCK) and LIM-kinase (LIMK) signaling [6, 7, 42, 47]. Cofilin phosphorylation and actin stability must be precisely regulated to maintain optimal dendritic spine turnover and stability, and are essential for normal spine/synaptic function and learning and memory; dysregulation in either direction can have detrimental effects [54, 70, 93]. Importantly, p75^NTR^ can also alter tau phosphorylation by modulating multiple tau kinases, including GSK3β, CDK5, JNK, Fyn, and p38, and by increasing RhoA activity, which promotes activation of the tau kinase ROCK [9, 40, 45, 56, 71, 72, 84, 92].

LM11A-31 is a small-molecule, orally bioavailable p75^NTR^ modulator that downregulates p75^NTR^-mediated degenerative signaling and upregulates survival signaling [48]. It blocks excess tau phosphorylation, tau mislocalization, and neuritic dystrophy induced by Aβ oligomers in vitro [90]. In APP mouse models, LM11A-31 inhibits excess tau phosphorylation and misfolding and reduces neurite degeneration [34, 56, 73]. In the P301S (PS19) tauopathy mouse model, oral administration of LM11A-31 reduced accumulation of pathological forms of tau and reversed dendritic spine loss [91]. A recent clinical trial in subjects with mild to moderate AD found that LM11A-31 is generally safe and tolerable, slows longitudinal increases in CSF biomarkers of pre- and post-synaptic degeneration, and slows cortical FDG-PET signal loss, a surrogate marker of synaptic function [69].

Clarifying mechanisms by which p75^NTR^ modulation confers synaptic resilience and reduces accumulation of pathological tau species may expand understanding of the effector pathways involved and thereby open up new avenues to therapeutic strategies modulating such processes. In pursuit of this goal, we identified and characterized a neuronal p75^NTR^-LIMK-cofilin signaling axis that can be pharmacologically modulated to enhance synaptic resilience and reduce pathological tau accumulation.

## Methods

### Reagents

Recombinant oTau was prepared using a previously established protocol [82]. Briefly, human recombinant tau (human tau-441, 2N4R isoform, expressed in E. coli, rPeptide, Bogart, GA) was dissolved in MES buffer (4-morpholineethanesulfonic acid hydrate, pH 6.5) to achieve a final concentration of 5 µM. Dithiothreitol (Sigma-Aldrich Corp. St Louis, MO) was added to a final concentration of 10 µM. The solution was incubated for 10 min at 55 °C. Subsequently, heparin (H19, Thermo Fisher Scientific, Waltham, MA) was added to the solution to a final concentration of 5 µM to induce aggregation. The solution was placed on a shaking incubator at a speed of 1000 rpm for 4 h at 37 °C.

The LIMK inhibitor BMS-3 and the slingshot phosphatase (SSH) inhibitor D3 were purchased from MedChemExpress LLC (Monmouth Junction, NJ).

LM11A-31 [(2S,3S)-2-amino-3-methyl-N-(2-morpholinoethyl) pentanamide], in a sulfate salt form, (MW: 243 in free base form, 439.34 in salt form) was custom-manufactured by Olon Ricerca Biosciences, achieving > 99% purity as confirmed by liquid chromatography-mass spectrometry analysis.

### Animals and treatment

All animal procedures complied with the National Institutes of Health Guide for the Care and Use of Laboratory Animals and were approved by the Institutional Animal Care and Use Committee at Stanford University. Timed pregnant C57BL/6 mice were purchased from Charles River Laboratories and delivered on embryonic day 15 (E15), after which E16 embryos were used for primary hippocampal cultures. Male transgenic mice expressing a human tau transgene with a P301S mutation (PS19) along with age- and strain-matched wild type (Wt) mice were purchased from The Jackson Laboratory (008169, B6;C3-Tg(Prnp-MAPT*P301S)PS19Vle/J). Wt and PS19 mice were administered either vehicle (sterile water) or LM11A-31 at 50 mg/kg (salt form), dissolved in sterile water (5 mg/ml), via oral gavage once daily, five days per week (Monday–Friday), for 3 months starting at 6 months of age. At 9 months of age, one hour after the final dose, mice were sacrificed and perfused with PBS, followed by brain tissue harvesting. Mice had access to food and water ad libitum and were group-housed on a 12-h light/dark cycle.

### Preparation of oTau-containing S1p fractions from hippocampal tissue

oTau-containing S1p fractions were derived from the hippocampus of 9-month-old PS19 tauopathy mice and age-matched Wt mice using a previously described procedure [28, 29]. In some experiments, the mice were treated with either vehicle or LM11A-31 for 3 months prior to extraction and fractionation. The mouse brains were not pooled. Briefly, frozen hippocampal tissues (100–150 mg) were weighed, and a 10× volume of homogenization buffer was added to homogenize brain tissue. The homogenization buffer consisted of TBS buffer (50 mM Tris, pH 8.0, 274 mM NaCl, 5 mM KCl) supplemented with protease and phosphatase inhibitor cocktails (Roche, cat# 05892791001 and cat# 04906837001). The homogenate was then centrifuged at 48,300×*g* for 20 min at 4 °C. The supernatant (S1) fractions were centrifuged a second time at 186,340×*g* at 4 °C for 40 min. The pellet (S1p) fraction was then resuspended in TE buffer at a volume (400–600 μl) that was 4× the starting weight of the tissue homogenate. The total protein concentration of the S1p fraction was measured by the Precision Red Advanced Protein Assay (Cytoskeleton, Inc., Denver, CO) and then adjusted to 4 mg/mL. The procedure of hippocampal tissue extraction, fractionation, and protein concentration adjustment of the S1p fraction involved an approximately 40× dilution. Samples were run on 4–12% SDS-PAGE gels, and blots were probed with the tau-13 antibody, which recognizes both normal and pathological conformations of tau, including oTau and full-length tau [13, 29]. Western blot analysis of the S1p fractions revealed bands primarily within the 100–150 kDa range, along with higher and lower molecular weight species (Supplementary Fig. 1), consistent with previous analyses showing that the S1p fraction is enriched in oTau, primarily dimers and trimers [28]. The concentration of total tau in S1p fractions was measured by comparison to a concentration curve generated from serial dilutions of recombinant tau analyzed by immunoblotting and quantitated with UN-SCAN-IT Gel & Graph Digitizing Software ver. 6.14 (Silk Scientific Inc., Orem, UT). The fractions were further diluted by approximately 100-fold with TE buffer to 40 μg/mL of tau for storage and future use. S1p fractions from Wt tissue were also diluted by 100-fold with TE buffer. A sarkosyl-insoluble fraction, primarily containing tau fibrils, could be generated from the discarded pellet from the first centrifugation step, but this was not done in the present study [28].

### Hippocampal neuron cultures and treatments

Primary embryonic mouse hippocampal neuron cultures were plated on poly-D-lysine-coated (10 µg/ml) glass cover slips at 40,000–50,000 cells per well in 12-well plates (Corning Life Sciences, Tewksbury, MA). Cells were seeded in plating medium, Dulbecco’s Modified Eagle Medium/Nutrient Mixture F-12 (DMEM/F12) supplemented with 10% fetal bovine serum and 1× penicillin/streptomycin (PS), for 2 h. The medium was then changed to neurobasal medium (Thermo Fisher Scientific) supplemented with PS, B27 (Thermo Fisher Scientific), and 2 mM glutamine as described previously [92]. After 21 days in culture under these conditions, murine neurons exhibit an adult-like phenotype with respect to neuronal dendritic spines and functional synapses [95]. At 20–21 days, cells were exposed to oTau at a final concentration of 50 nM for recombinant tau or 40 µg/ml for oTau within S1p fractions under conditions described in the figure legends and results section. All signaling pathway components were assessed following 1 h of treatment, and changes in pTau (using AT8 and pTau217 antibodies) were assessed following 3 h of treatment, as these changes are fairly rapid [92, 96]. Assessment of tau oligomerization with the T22 antibody was performed at 48 h of treatment, as performed in our previous work [92].

### Protein extraction and western blot analyses

Cultured hippocampal neurons were homogenized in RIPA lysis buffer (150 mM NaCl; 1% NP-40; 50 mM Tris, pH 7.4; 1 mM EDTA; 10% glycerol; 1 mM PMSF) with one tablet of complete proteinase inhibitor (Thermo Fisher Scientific, Cat# A32959) as described previously [91]. The homogenates were centrifuged at 10,000×*g* for 10 min, after which the supernatants were collected. Protein concentration was measured using the Precision Red Advanced Protein Assay (Cytoskeleton, Inc). All protein extracts were stored at − 80 °C for subsequent experimentation. For Western blotting, 20–40 µg aliquots of protein extracts from each sample were run in each lane on precast NuPAGE 4–12% Bis–Tris Gels for SDS-PAGE (Thermo Fisher Scientific) and then transferred to PVDF membranes. After blocking with 5% non-fat dried milk at room temperature for 1 h, membranes were probed overnight at 4 °C with one of the following antibodies: p-cofilin^Ser3^ (Cat#3313), cofilin (Cat#5175), p-LIMK1^Thr508^/LIMK2^Thr505^ (Cat#3841), LIMK1 (Cat#3842), p-JNK^Thr183/Tyr185^ (Cat#9251), JNK (Cat#9252), p-PKCα/β II^Thr638/641^ (Cat#9375, all diluted to 1:1000, Cell Signaling, Beverly, MA); Tau13 (Cat#sc-21796) and α-fodrin (Cat#sc-46696, both diluted to 1:1000; Santa Cruz Biotechnology, Santa Cruz, CA); actin (Cat#A5441, 1:10,000; Sigma, St Louis, MO). The secondary antibodies used were either horseradish peroxidase (HRP)-conjugated anti-rabbit IgG (1:10,000; Thermo Fisher Scientific) or anti-mouse IgG (1:10,000; DAKO). Bands were detected using enhanced chemiluminescence (ECL) reagent (GE Healthcare, Sunnyvale, CA) following the manufacturer’s protocol. Immunoreactive band densities were measured using UN-SCAN-IT gel software (ver. 6.14, Silk Scientific Inc.).

### Immunofluorescence

Cultured hippocampal neurons were fixed in 4% formaldehyde for 20 min, permeabilized for 6 min in 80% ice-cold methanol, and incubated with primary antibodies at 4 °C overnight. Primary antibodies used were rabbit polyclonal MAP2 antibody (1:1000; Cat# 8707, Cell Signaling) to stain dendrites and mouse monoclonal drebrin antibody (1:300; Cat# ADI-NBA-110, Enzo, Farmingdale, NY) to stain drebrin-positive spines. Mouse monoclonal phospho-tau antibody AT8 (Cat#MN1020, 1:1000) and rabbit polyclonal antibody pTau^Thr217^ (p-tau217, Cat#44–744. 1:800; Thermo Fisher Scientific) were used to measure tau phosphorylation, and rabbit polyclonal T22 (Cat#ABN454, 1:500; EMD Millipore, Burlington, MA) was used to measure the presence of oTau [38]. Secondary antibodies used were either donkey anti-rabbit or anti-mouse conjugated with either FITC or Cy3 (1:400; Jackson ImmunoResearch Laboratories, Inc. West Grove, PA).

### Confocal imaging, quantification, and data analysis

All images for this study were acquired and analyzed by an investigator blinded to the experimental conditions.

For imaging of dendrites and dendritic spines, cultured hippocampal neurons were imaged using an oil-immersion 63× objective on a Leica DM5500 confocal microscope, and the images were processed using LAS X viewer software (Leica). The neuronal cell bodies (soma) were stained with DAPI and imaged at a wavelength of 405 nm (blue channel); the neuronal dendrites stained with MAP2 were imaged at a wavelength of 488 nm (green channel); and the drebrin-labeled spines were imaged at a wavelength of 561 nm (red channel). Z-stack images were acquired at a 1024 × 1024 resolution, a zoom factor of 1.8, and a z-step size of 0.5 µm. The number of steps in each z-stack image was determined by the size of the neuron in the z-plane to ensure that the entirety of each neuron was captured. The areas to be imaged were systematically selected from the top-left region to the bottom-right region on the coverslip. For imaging, preference was given to fields of view that had isolated neurons versus clustered neurons. The position of each field was recorded for reference.

For quantification of drebrin-positive spines, the images were analyzed using Imaris 9.3 software. The ‘filaments’ option in Imaris was applied to manually trace along the dendrites of every neuron by placing starting and seed points at the beginning and along the length of the dendrites, respectively. The largest dendrite diameter (i.e., starting point) was set at a 9.00 µm threshold, and the thinnest dendrite diameter (i.e., seed point) was set at a 2.50 µm threshold. After manually tracing the dendrites, the spines along the dendrites were assessed using the automatic ‘spine detection’ function in Imaris. The thinnest spine diameter was set at a 0.299 µm threshold, and the maximum spine length was set at 2.00 µm. The spine density (number of spines/dendrite length) was then calculated for each neuron.

For quantification of dendrite degeneration, Neurolucida (MBF Biosciences, Williston, VT) in the manual mode was used to measure the total length of dendrites and dendritic complexity for each neuron. Complexity is calculated using the following formula: Complexity = [Sum of the terminal orders + Number of terminals] * [Total dendritic length / Number of primary dendrites]. Terminal is defined as endings, and terminal order is defined as the number of sibling branches encountered going from the terminal to the cell body [55, 60]. One neuron was imaged and quantified per field.

For measuring tau oligomers, we modified a previously described method [91] using the T22 oligomer-specific antibody. Images of cultured hippocampal neurons were acquired at ×40 magnification using a Leica DM5500 confocal microscope (Leica Microsystems Inc., Buffalo Grove, IL). Laser power, gain, and offset settings were optimized using LAS X viewer software (Leica) on samples with positive signals and then applied uniformly to all image files within a staining set, which included all three treatment conditions. Image files were converted to 8-bit grayscale using FIJI/ImageJ. Background subtraction was performed automatically using the rolling ball algorithm. Threshold ranges were visually selected for each staining set to optimize signal capture for both cell area and T22-positive particles. These threshold ranges were then consistently applied to all images in the set before performing the “Analyze Particles” function to quantify T22-positive particle area and total cell area per field. The ratio of total fluorescent T22-positive particle area to total cell area within each field was derived.

UN-SCAN-IT Gel & Graph Digitizing Software ver. 6.14 (Silk Scientific Inc.) was used to quantify total pixel intensity in immunofluorescent images. The average total pixel intensity per cell was calculated as the total level of fluorescent antibody-associated signal per field (AT8 pTau^S202/T205^, pTau^Y217^) divided by the total number of neurons in the field, and the data were normalized as a percentage of the mean value of the culture medium control.

### G-LISA RhoA activation assays

For analysis of RhoA activation, a G-LISA RhoA Activation Assay Biochem kit and a total RhoA ELISA kit (Cytoskeleton, Inc. Denver, CO) were used according to the manufacturer’s instructions. Briefly, protein concentrations in hippocampal protein lysates were measured using the Precision Red Advanced Protein Assay (Cytoskeleton, Inc) and normalized to the same concentration across all samples. Samples were then incubated in RhoA-GTP affinity plates for 30 min to capture activated RhoA, or in total RhoA affinity plates for 60 min to capture total RhoA. Captured activated and total RhoA was measured with RhoA-specific antibodies. RhoA activation was quantified as the ratio of activated RhoA to total RhoA within the same lysates. This ratio was then normalized to the mean value of the culture-medium control.

### Statistics

Data were analyzed for statistical significance using either GraphPad Prism software (version 10.2) or R (v4.3.3) [78]. The numbers of samples and measurements are indicated in the figure legends. For bar graphs quantifying spine density, dendrite health, and pathologic tau (AT8 and pTau217) comparisons were made using a linear model accounting for batch and group effects, followed by pairwise comparisons using estimated marginal means (emmeans v2.0.0) [39] with Bonferroni correction. Models were tested for normality of residuals (Shapiro–Wilk) and homogeneity of variance (Levene), and in absence of either, data were inverse rank normalized (inverse normal transformation, INT) (RNOmni v1.0.1.2) [49] and refit for non-parametric statistical testing via the same methods. For ease of interpretation, untransformed, batch-corrected data points are shown in figures via subtraction of the batch component of the model. For T22 antibody staining, data from the ratio of total T22-positive particle area to total cell area in each field were analyzed. T22 data was assessed via non-parametric testing with the Kruskal–Wallis test followed by Dunn’s post-hoc analysis. For Western blots, values were normalized to the corresponding culture-medium lane on each membrane. Data were assessed for normality using the Shapiro–Wilk and D’Agostino–Pearson tests. For normally distributed Western blot data, we used parametric, one-way analysis of variance (ANOVA) followed by Dunnett’s post-hoc analysis, and for non-normally distributed data, we used a non-parametric, Kruskal–Wallis test followed by Dunn’s post-hoc analysis. Cumulative frequency distributions were compared using the two-sample Kolmogorov–Smirnov (KS) test. The p-values are indicated within the graphs. The statistical methods for each experiment are further specified in the figure legends. All data were expressed as mean or estimated marginal mean ± standard error (SE).

## Results

### Modulation of p75^NTR^ mitigates neuronal degeneration induced by recombinant oTau and oTau-containing S1p fractions from PS19 mouse hippocampi

**Fig. 1:**
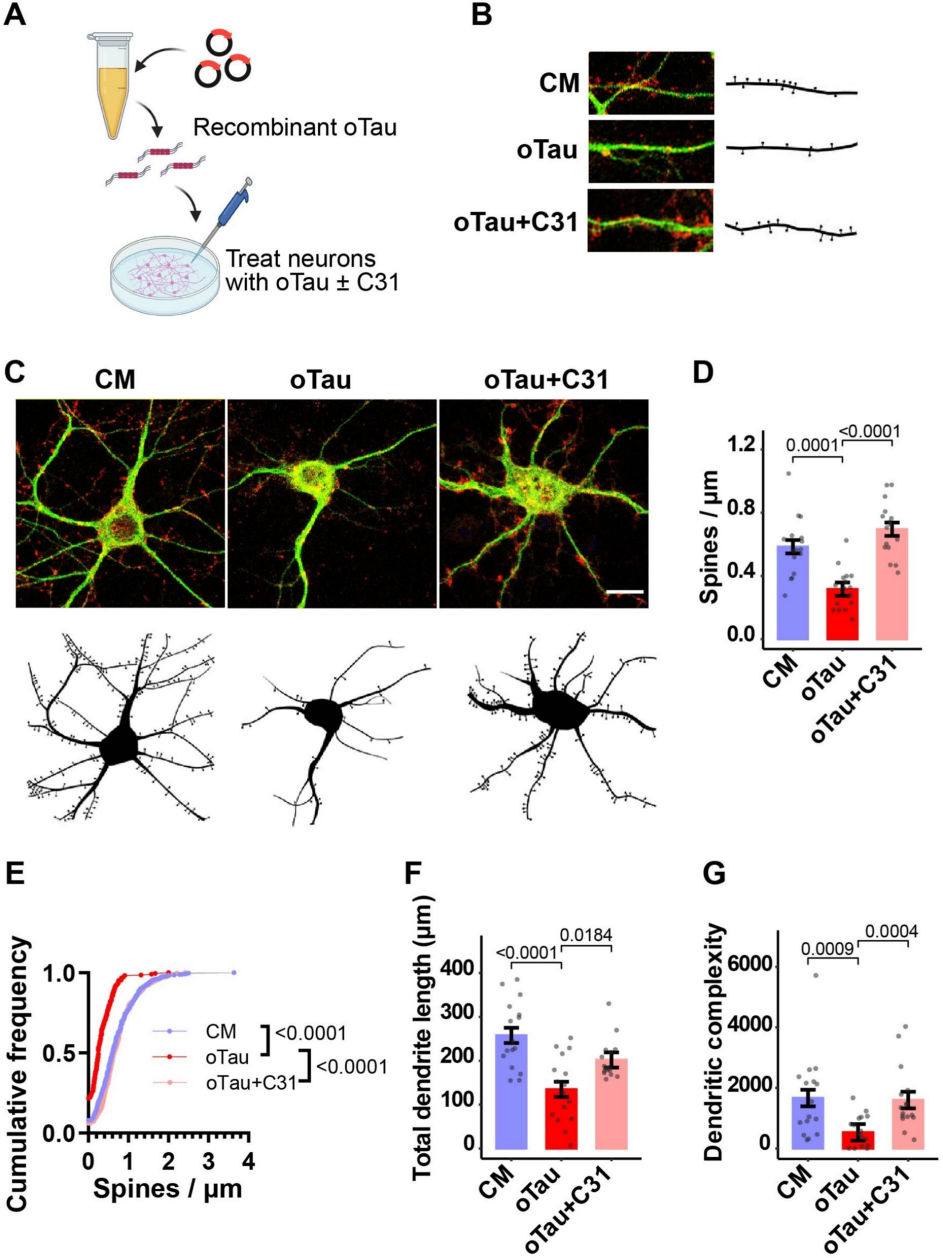
p75^NTR^ modulation prevents spine and dendrite degeneration in neurons exposed to recombinant oTau. **A** Schematic of experimental design. Created with BioRender.com. **A**–**G** Hippocampal neurons at 20 days in vitro were treated with culture medium (CM) or recombinant oTau (50 nM) ± concomitant LM11A-31 (C31, 100 nM). Neurons were fixed 24 h after treatment. Fixed neurons were stained for drebrin and MAP2. MAP2-positive dendrites were traced, and drebrin–positive spines were quantified using Neurolucida software. **B**, **C** Representative confocal images and corresponding Neurolucida tracings of **B** dendritic spines and **C** neuron-associated dendrites. **D**, **E** Quantitation of dendritic spine density (the number of spines per length of dendrite segment) displayed as **D** batch-corrected spines/µm and **E** cumulative frequency distribution. **F** Batch-corrected total dendrite length per neuron. **G** Batch-corrected dendritic complexity. All bars show estimated marginal mean ± SE. Statistical significance was determined using a linear model (for **D**, Spine density ~ group + batch, F(5, 42) = 14.503, *p* = 2.94e−08; for **F**, Total dendrite length ~ group + batch, F (5, 42) = 7.12, *p* = 6.68e−05; for **G,** INT Dendritic complexity ~ group + batch, F(5, 42) = 6.144, *p* = 2.34e−04) followed by pairwise comparison of estimated marginal means (Bonferroni p-adj shown in figure), or in **E** using Kolmogorov–Smirnov test. **D–G** n = 16 neurons per condition from a total of 4 independent experiments (n = 4 neurons per condition per experiment). INT–inverse normal transformation

The hippocampus is particularly vulnerable to neuronal and synaptic degeneration. oTau disrupts neuronal and synaptic function and contributes to the formation of neurofibrillary tangles [22, 37, 46, 81]. Extracellular oTau can be internalized by neurons and induce intracellular aggregation of endogenous tau in cultured primary cortical neurons, impair long-term potentiation in hippocampal slices, and nucleate tau aggregation and impair memory in vivo. In the present study, to characterize oTau effects in hippocampal neurons, cultures at 20 days in vitro (DIV) were exposed to recombinant oTau, stained for the dendritic spine marker drebrin and the neuronal dendrite marker MAP2 (Fig. 1A), and analyzed morphometrically. Recombinant oTau caused a significant ~ 50% decrease in spine density **(**Fig. 1B–D**)** and a pronounced leftward shift in the cumulative frequency plot of spine density **(**Fig. 1B, E**)**. Recombinant oTau also reduced total dendritic length and complexity, indicating dendrite degeneration **(**Fig. 1C, F, G**).** Addition of LM11A-31 concomitant with oTau preserved dendritic spine density and protected dendrites from degeneration (Fig. 1B–G). Thus, an oTau preparation generated from a bacterial source exhibited neuronal degenerative effects, which could be inhibited by LM11A-31.

**Fig. 2:**
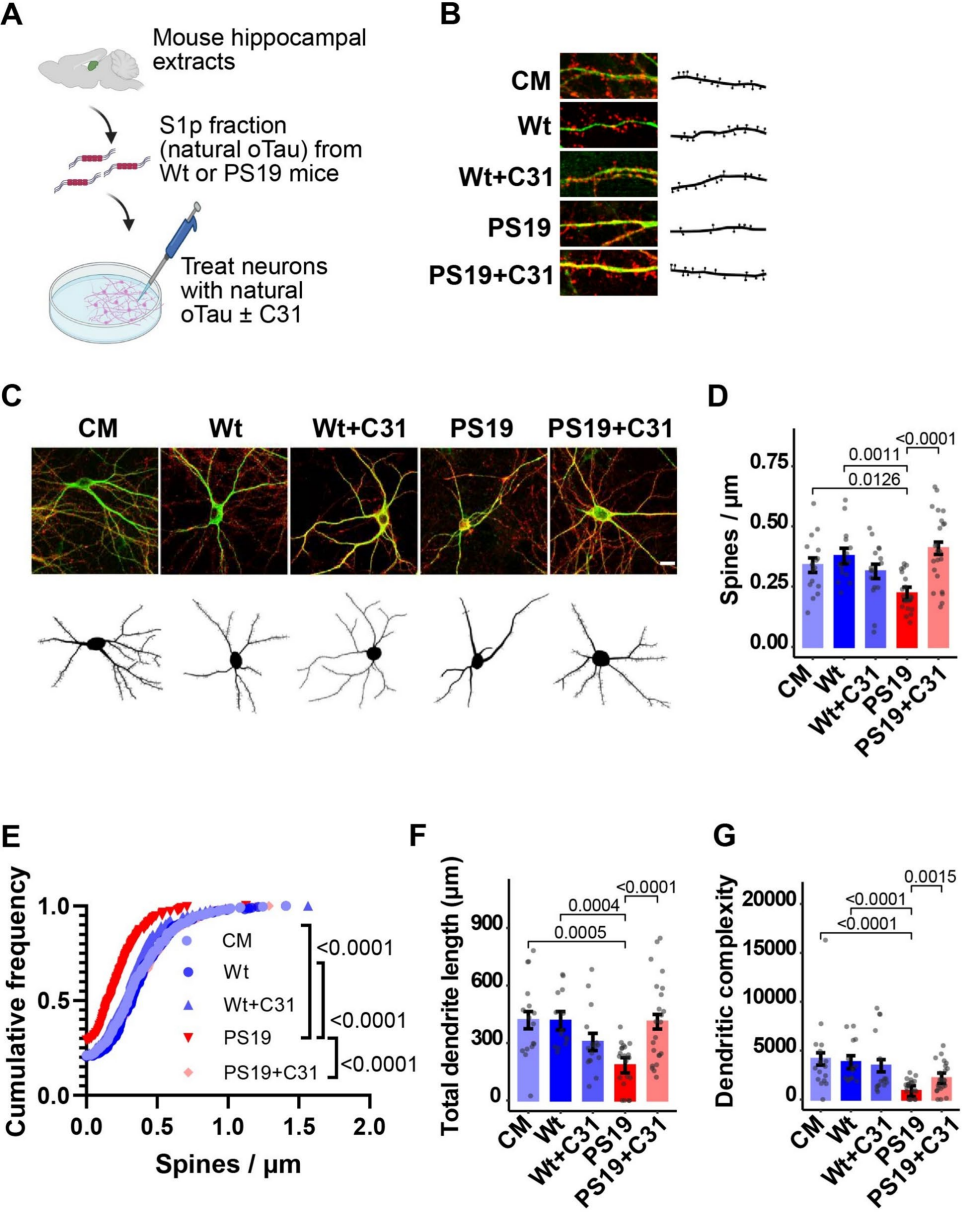
p75^NTR^ modulation prevents spine and dendrite degeneration in neurons exposed to oTau-containing S1p fractions from PS19 mice. **A** Schematic of experimental design. Created with BioRender.com. **A**–**G** Hippocampal neurons at 20 days in vitro were exposed to culture medium (CM) alone or to hippocampal S1p fractions (designed to capture oTau) from Wt or PS19 mice ± LM11A-31 (C31, 100 nM) added to the cultured neurons. Neurons were fixed 24 h after treatment. Fixed neurons were stained for drebrin and MAP2. MAP2-positive dendrites were traced, and drebrin-positive spines were quantified using Neurolucida software. **B**, **C** Representative confocal images and corresponding Neurolucida tracings of **B** dendritic spines and **C** neuron-associated dendrites. **D**, **E** Quantitation of drebrin-positive spine count per length of dendrite segment displayed as **D** batch-corrected spines/µm and **E** cumulative frequency distribution. **F** Batch-corrected total dendrite length per neuron. **G** Batch-corrected dendritic complexity. All bars show estimated marginal mean ± SE. Statistical significance was determined using a linear model (for **D**, Spine density ~ group + batch, F(6, 83) = 7.155, *p* = 3.61e−06; for **F**, INT Total dendrite length ~ group + batch, F (6, 83) = 10.523, *p* = 1.17e−08; for **G,** INT Dendritic complexity ~ group + batch, F(6, 83) = 10.392, *p* = 1.44e−08) followed by pairwise comparison of estimated marginal means (Bonferroni p-adj shown in figure), or in **E** using Kolmogorov–Smirnov testing. **D**–**G** n = 14–23 neurons per condition from a total of 3 independent experiments (n = 3–12 neurons per condition per experiment). INT–inverse normal transformation

Given that recombinant oTau may lack the full repertoire of post-translational modifications, three-dimensional conformations, and other degenerative properties present in naturally derived oligomers, we also examined LM11A-31 effects in neurons exposed to hippocampal tissue-derived ‘natural, non-recombinant’ oTau, which is enriched in S1p fractions (as described in Methods). Hippocampal neurons at 20 DIV were treated for 24 h with oTau-containing S1p fractions from PS19 mice or with control S1p fractions from age-matched Wt mice (Fig. 2A). Spine density decreased by approximately 44% in hippocampal neurons exposed to PS19 S1p fractions, but no decrease was detected with exposure to Wt S1p fractions, consistent with the presence of oTau in PS19 but not Wt fractions (Fig. S1 A–D). Concomitant treatment of cultures with LM11A-31 blocked spine density loss occurring in neurons exposed to PS19 S1p fractions but had no effect on neurons exposed to Wt S1p fractions **(**Fig. 2B–E**)**. Exposure of hippocampal neurons to PS19 S1p fractions caused a decrease in total dendrite length and complexity, while exposure to Wt S1p fractions did not cause dendritic degeneration. LM11A-31 treatment protected dendrites from degeneration caused by exposure to PS19 S1p fractions but had no effect on dendrites exposed to Wt S1p fractions (Fig. 2F, G). LM11A-31 preserved the structural integrity of hippocampal dendritic spines and dendrites when applied at the same time as extracellular oTau or S1p hippocampal extracts, suggesting that LM11A-31 rapidly promotes resilience to a range of pathogenic oTau species and perhaps other, non-tau factors.

### Chronic in vivo p75^NTR^ modulation reduces hippocampal S1p fraction toxicity

**Fig. 3:**
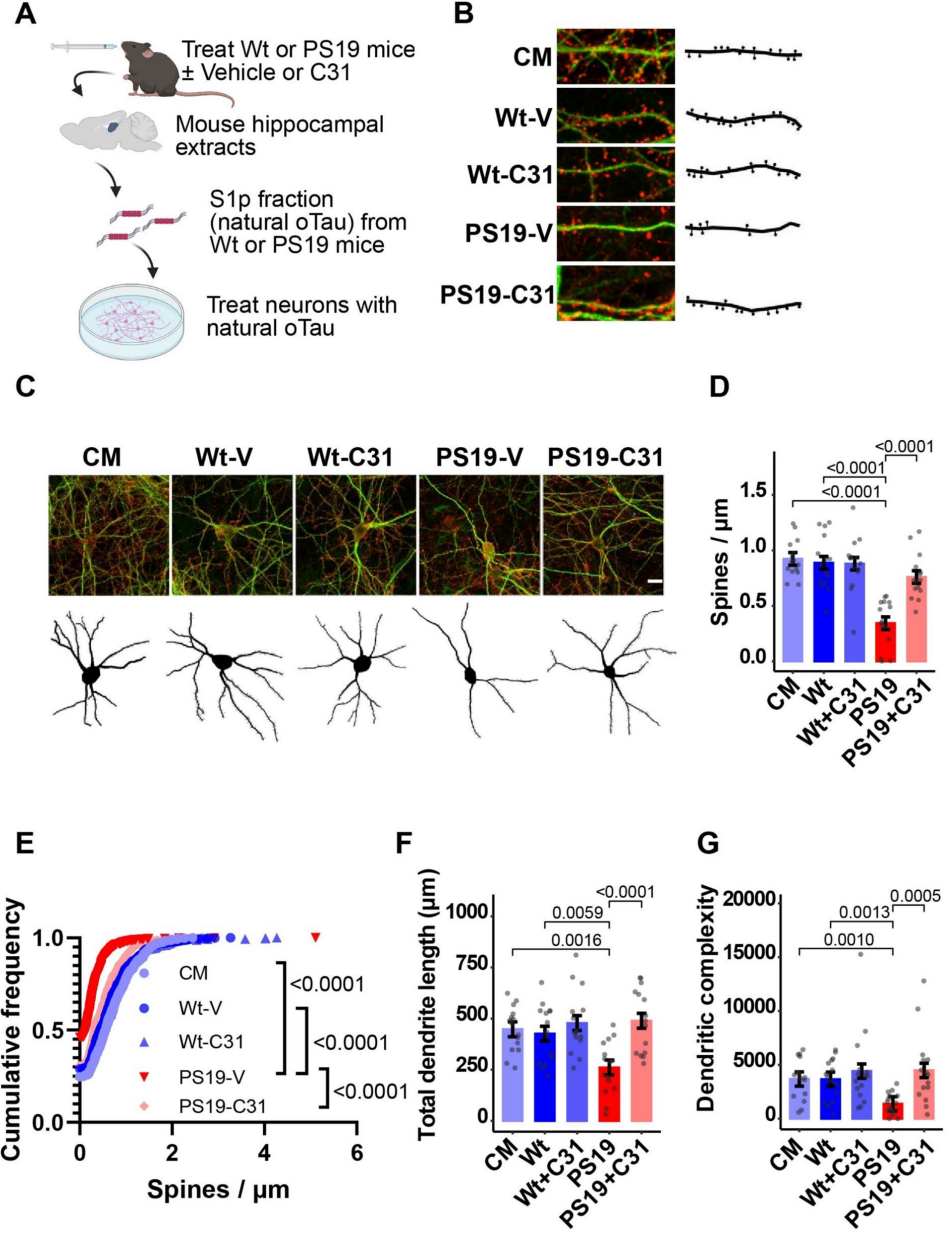
Spine and dendrite degeneration do not occur in cultured neurons exposed to S1p fractions from mice treated with LM11A-31. **A** Schematic of experimental design. Created with BioRender.com. **A**–**G** Hippocampal neurons at 20 days in vitro were exposed to culture medium (CM) alone or to S1p fractions (designed to capture oTau) from Wt or PS19 mice administered vehicle or LM11A-31 (C31). Neurons were fixed 24 h after treatment. Fixed neurons were stained for drebrin and MAP2. MAP2-positive dendrites were traced and drebrin-positive spines were quantified using Neurolucida software. **B**, **C** Representative confocal images and corresponding Neurolucida tracings of **B** dendritic spines and **C** neuron-associated dendrites. **D**, **E** Quantitation of dendritic spine density (drebrin-positive spine count per length of dendrite segment) displayed as a **D** batch-corrected spines/µm and **E** cumulative frequency distribution. **F** Batch-corrected total dendrite length per neuron. **G** Batch-corrected dendrite complexity. All bars show estimated marginal mean ± SE. Statistical significance was determined using a linear model (for **D**, Spine density ~ group + batch, F(6, 68) = 28.025, *p* < 2e−16; for **F**, Total dendrite length ~ group + batch, F (6, 68) = 7.172, *p* = 5.96e−06; for **G,** INT Dendritic complexity ~ group + batch, F(6, 68) = 4.001, *p* = 0.002) followed by pairwise comparison of estimated marginal means (Bonferroni p-adj shown in figure), or in **E** using Kolmogorov–Smirnov testing. **D**–**G** n = 15 neurons per condition from a total of 3 independent experiments (n = 5 neurons per condition per experiment). INT–inverse normal transformation

In a previous study, LM11A-31 reduced tau phosphorylation and ameliorated the loss of dendritic spines and dendritic complexity in hippocampal neurons of PS19 mice, and it reduced the accumulation of multiple post-translationally modified and aggregated tau forms [91]. These findings raised the possibility that treatment with LM11A-31 might reduce accumulation of pathogenic forms of tau that cause neuronal degeneration. To address this possibility, we investigated the effects of treating cultured hippocampal neurons with S1p fractions from vehicle-treated or LM11A-31-treated PS19 tauopathy mice. Given the possibility of carryover of active drug in brain extracts, we noted that the extraction and fractionation protocol includes the disposal of the aqueous fraction containing water-soluble LM11A-31 and multiple dilution steps, including a final 1:1000 dilution into culture media, resulting in a total dilution factor of approximately 1:4,000,000 and a final tau concentration of 40 ng/ml. Previously, brain fractions from mice treated under a similar protocol [34] exhibited an LM11A-31 concentration of 1900 nM. With the series of dilutions involved in preparation of S1p fractions, we estimate that the maximum final concentration of LM11A-31 in the culture media after treatment with S1p fractions is approximately 0.5 pM, which is ~ 40,000-fold below the EC50 of approximately 20 nM [90]. Thus, effects observed in cultured hippocampal neurons exposed to S1p fractions from LM11A-31-treated PS19 mice likely reflect drug-induced reduction of various pathogenic tau species [91] such as oTau in the mouse brain, or other neurodegenerative factors, rather than direct effects of trace amounts of LM11A-31 (Fig. 3A). S1p fractions from Wt mice treated with either vehicle or LM11A-31 did not induce changes in the morphometric measurements (Fig. 3B–G). In contrast, exposure to S1p fractions from vehicle-treated PS19 mice caused a significant reduction in spine density, total dendrite length, and dendritic complexity. Neurodegenerative effects of S1p fractions from PS19 mice treated with LM11A-31 were significantly decreased. There remained a small residual degenerative effect on spine density while effects on dendrite length and complexity were fully inhibited. The protection of neurons exposed to S1p fractions from PS19 mice treated with LM11A-31 is consistent with a drug-induced reduction of tissue factors promoting degeneration. Western blot analysis of S1p fractions isolated from Wt and PS19 mice treated with or without LM11A-31 demonstrated that LM11A-31 reduces levels of pathological tau (Fig. S1A–D). These findings are consistent with our previous observation that treating PS19 mice with LM11A-31 lowers levels of several potentially pathogenic oligomeric tau species in hippocampal lysates [91], and one or more of these are strong candidates for the toxic factors relevant to the observed effects, though their precise identity remains to be determined.

### Modulation of p75^NTR^ reduces accumulation of pathogenic tau species in hippocampal neurons in vitro

**Fig. 4:**
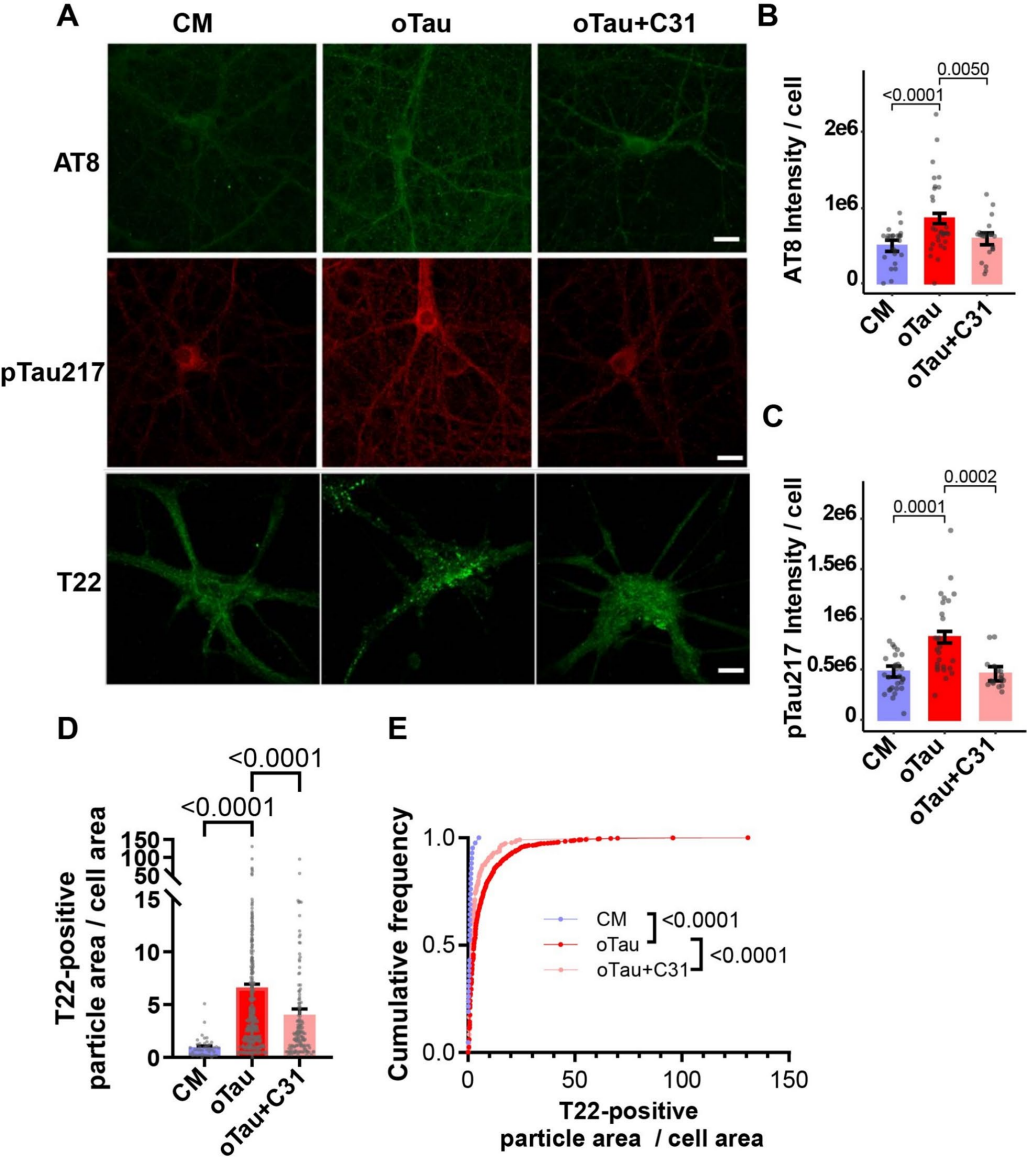
p75^NTR^ modulation inhibits recombinant oTau-induced tau phosphorylation and aggregation. **A**–**E** Hippocampal neurons at 20–21 days in vitro were exposed to CM or recombinant oTau ± concomitant LM11A-31 (C31, 100 nM) and immunostained with the indicated antibodies. **A** Representative images of immunostaining for AT8, pTau^Thr217^, and T22 (an oTau-specific antibody) following treatment durations of 3 h (AT8, pTau^Thr217^) or 48 h (T22). **B**, **C** Batch-corrected tau phosphorylation (AT8 and pTau^Thr217^ staining) as ratios of total fluorescence intensity to total cell number per field. Bars show estimated marginal mean ± SE from **B** n = 23–30 neurons per condition from a total of 3 independent experiments (n = 6–11 neurons per condition per experiment) and **C** n = 17–29 neurons per condition from a total of 3 independent experiments (n = 3–13 neurons per condition per experiment). Statistical significance was determined using a linear model (for **B**, INT AT8 intensity ~ group + batch, F(4, 73) = 54.761, *p* < 2e−16; for **C**, INT pTau217 intensity ~ group + batch, F (4, 67) = 12.134, *p* = 1.78e−07) followed by pairwise comparison of estimated marginal means (Bonferroni p-adj shown in figure). **D**, **E** Tau oligomers quantified as the ratio of total T22-positive particle area to total cell area in each field. Data are displayed as a **D** bar graph using the Kruskal–Wallis test with Dunn’s multiple comparisons test (H = 72.77, *p* < 0.0001), and **E** cumulative frequency distribution using Kolmogorov–Smirnov testing. **D**, **E** Area of T22-positive particles was measured in n = 18–19 neurons per condition from a total of 3 independent experiments (n = 6–7 neurons per condition per experiment), mean ± SE. INT–inverse normal transformation

Tau modifications such as phosphorylation, cleavage, misfolding, and missorting have been proposed to play a role in the degeneration of spines and dendrites in AD and other tauopathies [3, 66, 81]. Given the known effects of p75^NTR^ modulation on multiple tau kinases, including CDK5, GSK3β, JNK, and ROCK [45, 62, 90, 91], we hypothesized that LM11A-31 would inhibit intraneuronal accumulation of hyperphosphorylated tau and oTau induced by oTau exposure. To test this, we treated hippocampal neurons with recombinant oTau and then immunostained with the AT8 antibody for tau phosphorylated at Ser202 and Thr205 (pTau^Ser202/Thr205^), which is associated with enhanced tau polymerization and aggregation [16, 64]. Exposure of hippocampal neurons to recombinant oTau increased the levels of AT8 signal compared to untreated neurons, and concomitant treatment with LM11A-31 reduced this effect (Fig. 4A, B). Tau phosphorylated at threonine 217 (pTau^Thr217^), recently described as a biomarker for accumulation of amyloid and tau pathology [4, 5], increased after neurons were exposed to recombinant oTau, and LM11A-31 blocked this increase (Fig. 4A, C). Tau hyperphosphorylation promotes the production of oTau [20]. Since LM11A-31 reduced excess levels of tau phosphorylation, we asked whether it would also reduce tau oligomerization in neurons treated with recombinant oTau. oTau can be detected with the T22 antibody [38]. With exposure to recombinant oTau, total T22-positive particle area/cell area increased significantly. Notably, these effects were significantly diminished by the addition of LM11A-31 (Fig. 4A, D, E).

**Fig. 5:**
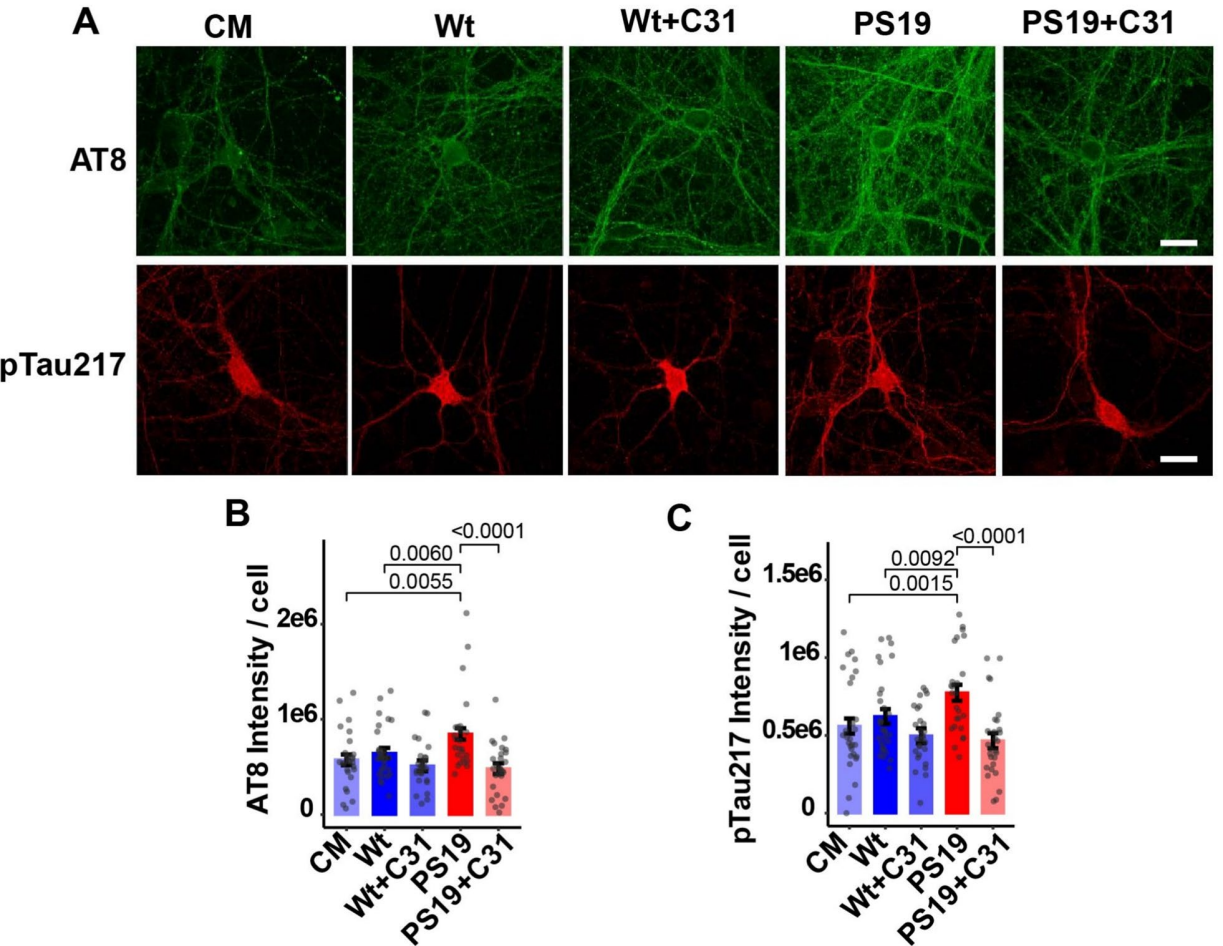
p75^NTR^ modulation inhibits oTau-containing S1p fraction-induced tau phosphorylation. **A**–**C** Hippocampal neurons at 21 days in vitro were treated with culture medium (CM) alone or with hippocampal S1p fractions (designed to capture oTau) from Wt or PS19 mice ± LM11A-31 (100 nM) added to the cultured neurons. Neurons were fixed 3 h following treatment. **B**, **C** Batch-corrected tau phosphorylation (AT8 or pTau^Thr217^ staining) as ratios of total fluorescence intensity to total cell number per field. Bars show estimated marginal mean ± SE from n = 25–30 neurons per condition from a total of 3 independent experiments (n = 5–12 neurons per condition per experiment). Statistical significance was determined using a linear model (for **B**, INT AT8 intensity ~ group + batch, F(6, 132) = 51.156, *p* < 2e−16; for **C**, INT pTau217 intensity ~ group + batch, F (6, 132) = 25.176, *p* < 2e−16) followed by pairwise comparison of estimated marginal means (Bonferroni p-adj shown in figure). INT–inverse normal transformation

Given the potential differences between recombinant tau and brain-derived tau species, we examined the effects of hippocampal S1p fractions and LM11A-31 on the generation of phospho-tau forms in hippocampal neurons. Tau phosphorylation levels in neurons exposed to Wt S1p fractions were similar to levels observed in neurons treated with culture media alone. In contrast, levels of pTau^Ser202/Thr205^ and pTau^Thr217^ were significantly increased in neurons exposed to oTau-containing PS19 S1p fractions relative to neurons exposed to Wt S1p fractions or culture media alone. Co-treatment with LM11A-31 prevented excess pTau^Ser202/Thr205^ and pTau^Thr217^ in neurons exposed to PS19 S1p fractions, matching levels observed in neurons exposed to Wt S1p fractions or culture media alone (Fig. 5A–C). Thus, tau phosphorylation responses to recombinant oTau and PS19 hippocampal S1p fractions were similar and strongly suppressed by LM11A-31.

### p75^NTR^ modulation reduces oTau-induced alterations in neuronal structural homeostatic signaling in primary hippocampal neurons

**Fig. 6:**
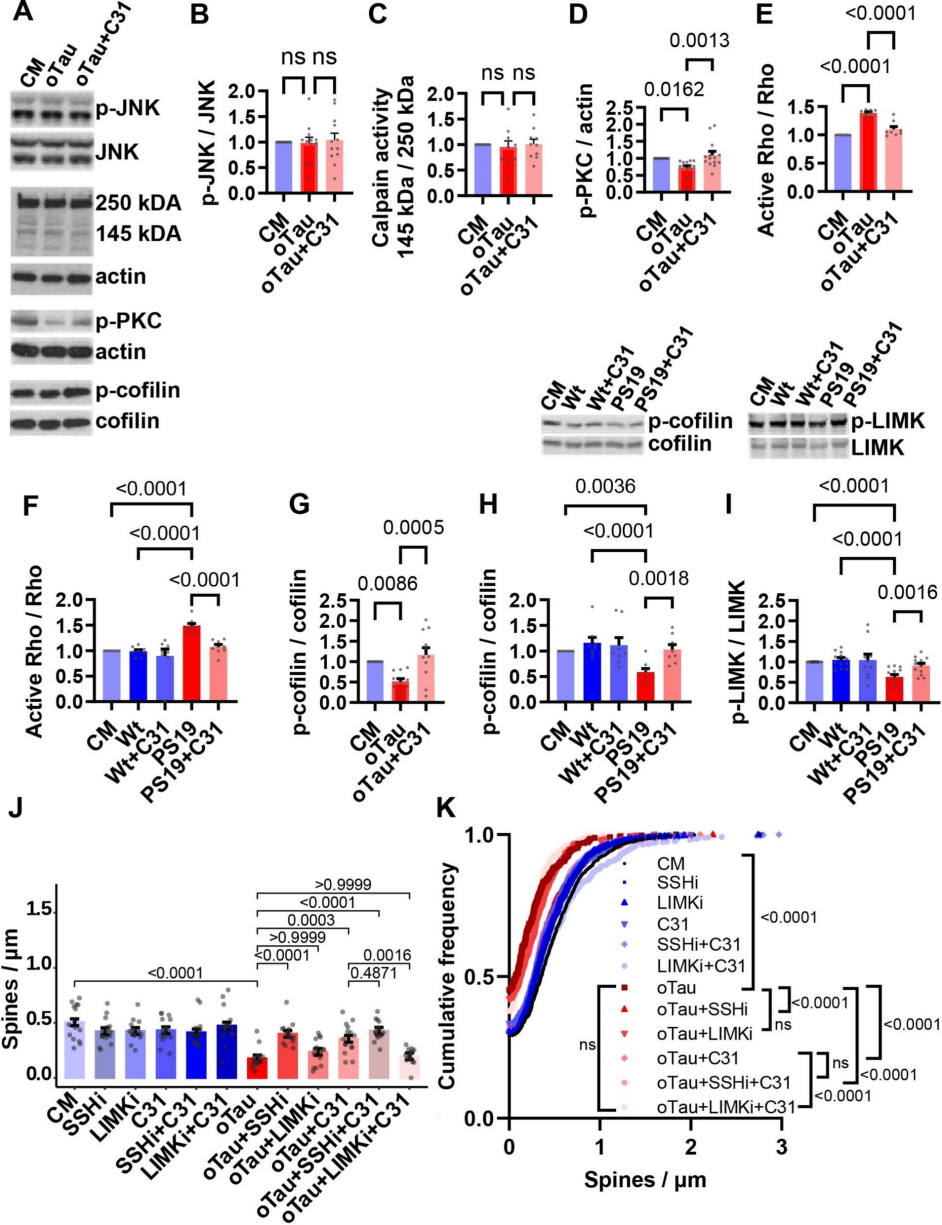
p75^NTR^ modulation mitigates oTau-induced alterations in PKC, RhoA, LIMK1, and cofilin signaling. **A**–**I** Hippocampal neurons at 21 days in vitro were collected one hour after the indicated treatments. **A**–**E**, **G** Neurons were treated with culture medium (CM) or recombinant oTau ± concomitant LM11A-31 (C31, 100 nM). **F**, **H**, **I** Neurons were treated with CM or hippocampal S1p fractions (designed to capture oTau) from Wt or PS19 mice ± LM11A-31 (100 nM) concomitantly added to the cultured neurons. **A** Representative Western blot images are shown (**B**–**D**, **G**–**I**). Western blots were quantitated as ratios of phospho (p)-protein to total protein or actin; or for calpain activity, as a ratio of cleaved (~ 145 kDa) to uncleaved (~ 250 kDa) α-fodrin. All values were subsequently normalized to the CM condition. **E**, **F** Activation of RhoA was measured using a G-LISA assay kit. Statistical significance was assessed in **E**, **F** using Kruskal–Wallis testing with Dunn’s multiple comparisons test, for E, H = 17.36, *p* = 0.0002; for F, H = 21.18, p = 0.0003, n = 8 protein preparations from 8 independent experiments; in **B**–**D** and** G** using ordinary one-way ANOVA with Dunnett’s multiple comparisons testing (JNK, F (2, 33) = 0.08885, *p* = 0.9152) and Kruskal–Wallis testing with Dunn’s multiple comparisons [(calpain (H = 0.5687, *p* = 0.7458), PKC (H = 19.76, *p* < 0.0001), and cofilin (H = 14.49, *p* = 0.0007)]**,** n = 13 individual protein preparations for each condition from 13 independent experiments; and in **H**, using Kruskal–Wallis with Dunn’s multiple comparisons test (H = 14.99, *p* = 0.0018), n = 8 individual protein preparations for each condition from 8 independent experiments; in **I** using Kruskal–Wallis testing with Dunn’s multiple comparisons test (H = 19.52, *p* = 0.0006), n = 10 individual protein preparations for each condition from 10 independent experiments. **J**, **K** Hippocampal neurons at 20 days in vitro were treated with CM or the slingshot inhibitor (SSHi) D3 (5 µM), or the LIMK inhibitor (LIMKi) BMS-3 (5 nM) ± oTau and/or ± C31 (100 nM). Neurons were fixed 24 h following treatment. Quantitation of dendritic spine density (number of spines per length of dendrite segment) displayed as a **J** batch-corrected spines/µm and **K** cumulative frequency distribution. Bars in **B**–**I** represent mean ± SE. Bars in **J** show estimated marginal mean ± SE. Statistical significance was determined using a linear model (for **J**, INT Spine density ~ group + batch, F(13, 166) = 14.279, *p* < 2e−16) followed by pairwise comparison of estimated marginal means (Bonferroni p-adj shown in figure), or in **K** using Kolmogorov–Smirnov testing of indicated comparisons. **J**, **K** n = 15 neurons per condition from 3 independent experiments (n = 3–6 neurons per condition per experiment). INT–inverse normal transformation

Previous studies have shown that mouse models expressing mutant human tau exhibit higher baseline activation of tau kinases, including JNK and CDK5 [68, 94]. Furthermore, p75^NTR^ signaling modulates JNK activation [84], and LM11A-31 reduces excess activation of JNK and the CDK5 activator calpain in vivo [91]. In contrast, addition of oTau to cultured hippocampal neurons did not increase the ratio of p-JNK^Thr183/Tyr185^/total JNK, an indicator of JNK activation. Moreover, LM11A-31 had no effect on JNK phosphorylation (Figs. 6A, B, S2). Activated calpain cleaves the 250 kDa cytoskeletal protein α-fodrin to fragments of ~ 145 kDa; the ratio of cleaved to full-length protein reflects calpain activity. We found that α-fodrin cleavage did not change after hippocampal neurons were exposed to recombinant oTau with or without LM11A-31 (Figs. 6A, C, S2).

PKC activation restores mature dendritic spines and memory in aged rats [23] and prevents synapse loss and memory deficits in mouse models of AD [19]. p75^NTR^ may have indirect effects on the activity of PKC [44, 87]. PKC activity can be regulated by phosphorylation, including autophosphorylation at Thr^638/641^, which promotes its activation [32]. We found that phosphorylation of PKC at Thr^638/641^ decreased in hippocampal neurons exposed to recombinant oTau; LM11A-31 blocked this effect (Figs. 6A, D, S2). In association with the finding that LM11A-31 prevents loss of dendritic spines, this finding suggests that reduction of oTau-induced PKC activation is a candidate mechanism contributing to LM11A-31 protective effects.

**Fig. 7:**
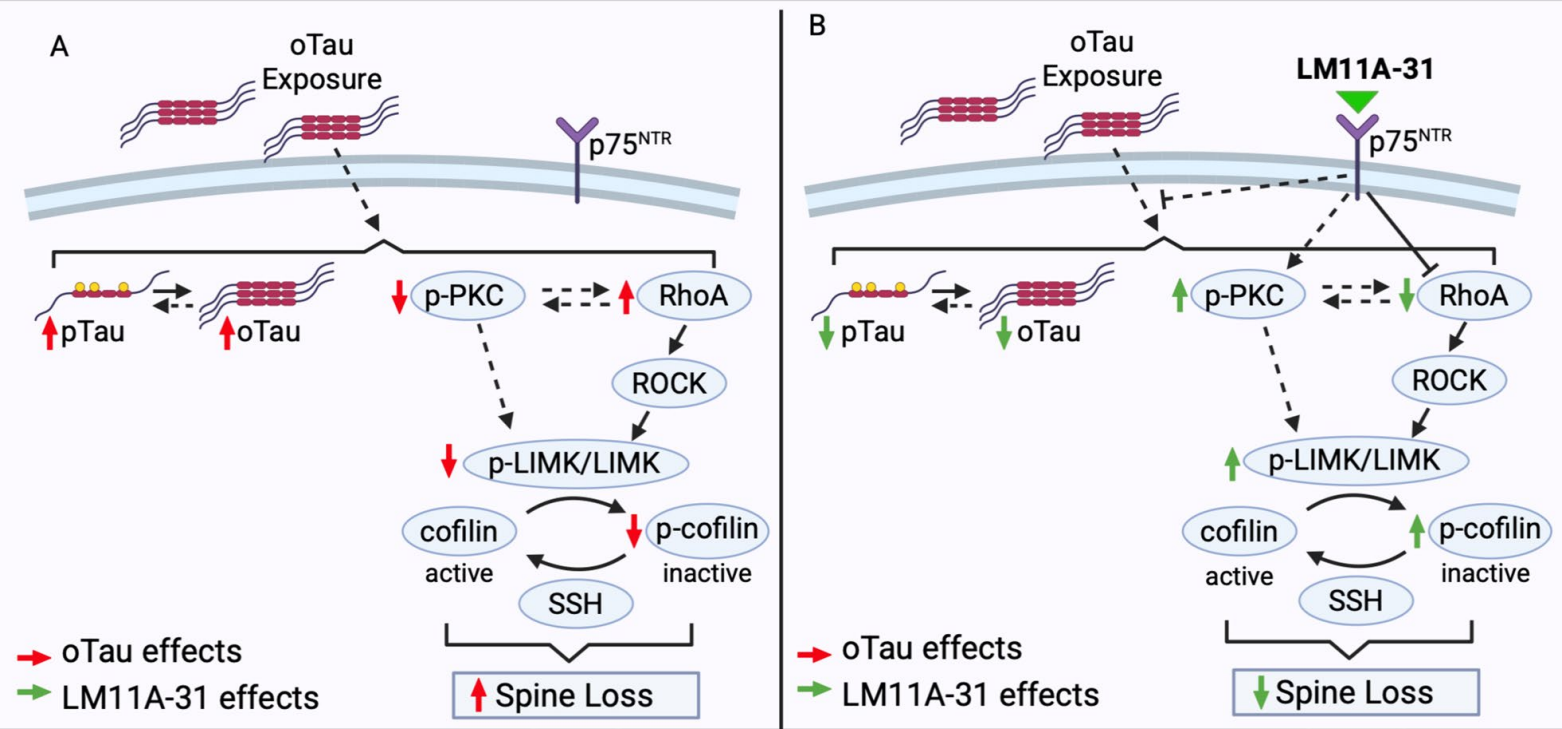
Proposed model of effects of p75^NTR^ modulation on oTau-induced accumulation of pathological tau, spine-related signaling, and dendritic spine loss. **A** oTau treatment induces tau phosphorylation, tau oligomerization, and excess RhoA activation. oTau treatment reduces PKC and LIMK phosphorylation/activity, and decreases cofilin phosphorylation, contributing to spine and dendrite degeneration. Solid black arrows indicate direct regulation, dashed arrows indicate indirect regulation. Red arrows indicate oTau effects. **B** LM11A-31 modulation of p75^NTR^ mitigates oTau-induced tau phosphorylation and oligomerization, and reduces oTau-induced alterations in RhoA-ROCK-LIMK/SSH-cofilin signaling, protecting the neurons against oTau-induced dendritic spine loss. Green arrows indicate LM11A-31 action

RhoA GTPase (RhoA) is a known regulator of actin cytoskeleton dynamics and synaptic spine stability [30]. Its activity can be directly modulated by p75^NTR^ [41, 88], and it can be excessively activated by pathogenic tau species [24]. Consequently, the protective effect of LM11A-31 on spine density in oTau-treated hippocampal neurons might involve inhibition of oTau-induced excess RhoA activation. Consistent with this, RhoA activation increased in hippocampal neurons treated with recombinant oTau (Fig. 6E) or oTau-containing S1p fractions from PS19 mice (Fig. 6F). Treatment of cultures with LM11A-31 prevented excess RhoA activation induced by both oTau- **(**Fig. 6E**)** and oTau-containing S1p fractions from PS19 mice (Fig. 6F). The alterations observed in RhoA activation prompted us to evaluate changes in downstream RhoA-mediated signaling pathways. RhoA signaling influences dendritic spine density through a well-characterized pathway (Fig. 7) in which RhoA activation results in ROCK-mediated phosphorylation and activation of LIMK**.** Active (phosphorylated) LIMK phosphorylates and inactivates cofilin, leading to actin stabilization [6, 7, 42, 47]. The phosphatase slingshot (SSH) dephosphorylates/activates cofilin, opposing LIMK action [30]. Cofilin activation promotes depolymerization of actin filaments, resulting in destabilization of dendritic spines [54]. Genetic reduction of cofilin mitigates spine loss and synaptic defects in Tau-P301S mice [85]. To investigate the effect of oTau on the activities of LIMK and cofilin, we conducted western blot analyses for cofilin and LIMK phosphorylation. Hippocampal neurons exposed to recombinant oTau (Figs. 6A, G, S2) or oTau-containing PS19 S1p fractions (Figs. 6H, S2) exhibited decreased phosphorylation of cofilin at the Ser3 site, indicating increased cofilin activation. Further, hippocampal neurons exposed to oTau-containing PS19 S1p fractions exhibited reduced phosphorylation of LIMK at Thr505/508, indicating reduced LIMK activation (Figs. 6I, S2). These results are consistent with previous studies showing increased activation of cofilin in a mouse model of tauopathy [91]. LM11A-31 treatment normalized cofilin Ser3 phosphorylation in the presence of recombinant oTau (Figs. 6A, G, S2) and in the presence of oTau-containing PS19 S1p fractions (Figs. 6H, S2), and normalized LIMK phosphorylation in the presence of oTau-containing PS19 S1p fractions (Figs. 6I, S2). LM11A-31 treatment did not affect cofilin or LIMK phosphorylation in neurons exposed to Wt S1p fractions (Figs. 6H, I, S2). Together, these results show that oTau treatment alters RhoA/LIMK/cofilin signaling, potentially destabilizing neuritic spines and synapses. LM11A-31 normalizes these signaling aberrations, suggesting a candidate mechanism of action underlying its ability to protect against oTau-induced dendritic spine degeneration.

Given that LM11A-31 inhibits oTau-induced dysregulation of LIMK and cofilin phosphorylation (Figs. 6G–I, S2) and oTau-induced loss of dendritic spine density (Figs. 1, 2, 3), we hypothesized that the effect of LM11A-31 on dendritic spine density depends on LIMK or SSH activity. We applied the LIMK inhibitor BMS-3 (which inhibits LIMK1 and LIMK2) [65] or the SSH inhibitor D3 to cultured hippocampal neurons that were exposed to recombinant oTau in the presence or absence of LM11A-31, and measured spine densities. Of note, in the absence of oTau, LIMK and SSH inhibitors had no discernible impact on spine density (Fig. 6J, K). Treatment with oTau resulted in a loss of spine density that was not affected by treatment with the LIMK inhibitor BMS-3 (Fig. 6J, K). LM11A-31 treatment prevented oTau-induced loss of spine density; however, in the presence of the LIMK inhibitor, LM11A-31 failed to prevent that loss (Fig. 6J, K). This finding suggests that under these conditions, preservation of dendritic spine density by LM11A-31 is dependent on LIMK activity. LIMK action is reversed by the cofilin phosphatase SSH. Together, they regulate the balance between phosphorylated (inactive) and dephosphorylated (active) cofilin [30, 52]. SSH inhibitor treatment prevented the dendritic spine loss induced by oTau, supporting the model that pharmacologically altering the balance of LIMK and SSH signaling in a way that should promote cofilin phosphorylation (inactivation) is beneficial to dendritic spine density (Fig. 6J, K).

## Discussion

In prior studies, we found that modulation of p75^NTR^ signaling with LM11A-31 reduced accumulation of high-molecular-weight forms of tau and restored hippocampal neuron dendritic spine density in PS19 tauopathy mice [91]. However, it remained unclear whether the effects on spine density resulted from reduced accumulation of pathological tau, enhanced resilience to pathological tau, or both. Our present findings indicate that LM11A-31 exerts a dual-protective effect by both reducing accumulation of degeneration-promoting tau species and preventing dendritic spine and dendrite degeneration triggered by exposure to pathogenic tau. These processes are illustrated in Fig. 7. Further, this study identifies molecular mechanisms underlying these effects, revealing additional potential targets for improving synaptic resilience and reducing pathological tau.

p75^NTR^ modulation diminished the accumulation of pTau and oTau **(**Fig. 7**)**. p75^NTR^ modulates multiple downstream pathways, some of which involve tau kinases, including GSK3β, CDK5, JNK, and ROCK [45, 62, 90, 91]. In prior studies, we found that targeting p75^NTR^ signaling with LM11A-31 inhibited tau-induced excess activation of enzymes that promote various pathological tau post-translational modifications [91]. Excessive RhoA and cofilin activation have been associated with downstream tau pathology [50, 85], thus LM11A-31 mitigation of excess cofilin activation might further contribute to reduced pathological tau accumulation. Consistent with these findings, genetic reduction of cofilin diminishes tau pathology in Tau-P301S mice [85].

In addition to reducing accumulation of pathological forms of tau, LM11A-31 also promoted resilience or protection against pathogenic tau species. p75^NTR^ modulates RhoA GTPase signaling [41, 88], which regulates actin dynamics and dendritic spine stability [31]. We observed that oTau treatment resulted in excessive RhoA activation and cofilin dephosphorylation (activation) in cultured hippocampal neurons, which was prevented by treatment with LM11A-31. Under normal physiological conditions, activated RhoA signals through ROCK to activate LIMK. Activated LIMK then phosphorylates (inactivates) the actin-depolymerizing protein cofilin, resulting in stabilization of actin filaments **(**Fig. 7**)** [6, 7, 42, 47]. SSH directly reverses the action of LIMK, thus promoting actin polymerization, stabilizing the actin cytoskeleton, and promoting maintenance of dendritic spines (Fig. 7**)** [30]. In our model of tau pathogenesis, excess RhoA activation occurred in tandem with LIMK inactivation and cofilin activation. This decoupling of RhoA activation and LIMK phosphorylation has been reported previously in aged mice [83] and suggests that oTau may disrupt additional regulators of cofilin activation. One such candidate is PKC, which interacts directly with RhoA [59] and mediates p75^NTR^-controlled RhoA activation [17, 63]. Concurrent with dendritic spine loss, oTau treatment reduced activation of PKC, an effect that was prevented by LM11A-31. In addition to modulating RhoA, PKC can promote Rac1 activation, which is known to induce LIMK activation [8, 80]. PKC can also interact directly with LIM domain-containing proteins such as LIMK, promoting phosphorylation (activation) [35]. Overall, our data support a model in which LM11A-31 preserves PKC and LIMK activity, thus preventing oTau-induced cofilin activation, actin destabilization, and loss of dendritic spines (Fig. 7B).

Amyloid pathology, another key pathological hallmark of AD, can promote aberrant RhoA/LIMK/cofilin signaling, contributing to dendritic spine degeneration and tau pathology [1, 11, 50, 67]. Cofilin activation is increased in AD patient brain tissue and AD mouse models [33, 51, 86], and treatment of hippocampal neurons with Aβ results in cofilin dephosphorylation (activation) [51]. Interestingly, actin and activated cofilin can form synaptotoxic rods and aggregates that have been shown to accumulate in the brains of AD patients, correlating with the extent of tau pathology [58, 61]. Activated cofilin may exacerbate tau pathology by competing with tau for microtubule binding [85]. Inhibiting the various degenerative signaling mechanisms described here, through p75^NTR^ modulation, may offer a therapeutic strategy for both primary and secondary tauopathies by reducing pathological tau accumulation and enhancing synaptic resilience to pathological tau.

Several limitations should be considered when interpreting these findings. The recombinant oTau used in our experiments lacks the full structural and post-translational complexity of brain-derived tau. We addressed this by exposing neurons to brain-derived oTau present in hippocampal extract fractions from PS19 mice expressing the human tau transgene with a P301S mutation, found in frontotemporal dementia. However, the formation—hence the molecular structure—of pathologic tau species resulting from this mutation may occur differently in the mouse brain compared to the human brain. In addition, the pathologic tau accumulating in these mice may differ from that occurring in tauopathies driven by other mutations or sporadic tauopathies [36]. We used male PS19 mice in our study because the degree of tau pathology and abnormal phenotype exhibited by female mice is mild relative to that of males and limits the ability to detect therapeutic effects [36, 74, 89]. Future studies are needed to determine whether LM11A-31’s ability to lower tau in vivo, and its protective effects on neurons exposed to oTau-containing extract fractions, also hold true when using extracts from different tauopathy models and from both male and female subjects. Additionally, identifying specific tau species within brain fractions that cause degeneration and determining which are affected by treatment with LM11A-31 will further clarify underlying mechanisms accounting for LM11A-31 therapeutic effects. Finally, our experiments do not distinguish whether the increase in intracellular tau after oTau exposure arises from neuronal uptake of exogenous oTau or from the induction and aggregation of endogenous tau. Both mechanisms are plausible. Future studies using labeled oTau, species-specific antibodies, tau-knockout neurons, or approaches that block tau synthesis or endocytosis will be required to define relative contributions.

## Conclusions

Our study suggests that p75^NTR^ modulation protects spines and dendrites by preventing accumulation of pathologically hyperphosphorylated tau and by normalizing RhoA/PKC/LIMK/cofilin signaling. These findings support p75^NTR^ modulation as a multi-mechanism strategy that promotes synaptic resilience and lowers pathological tau, supporting its promise as a treatment for AD and other tauopathies and identifying the p75^NTR^-LIMK-cofilin signaling axis as an additional potential target for promoting such effects.

## Supplementary Information

Additional file1 (DOCX 573 KB)

## Data Availability

The datasets generated during and/or analyzed during the current study are available from the corresponding author on reasonable request.

## Abbreviations

Aβ: Amyloid beta
AD: Alzheimer’s disease
oTau: Oligomeric Tau
p75^NTR^: P75 neurotrophin receptor
C31: LM11A-31
RhoA: Ras homolog family member A
PKC: Protein kinase C
LIMK1: LIM kinase 1
PI3K: Phosphoinositide 3-kinase
Ikk: IκB kinase
NFkB: Nuclear factor κB
ROCK: Rho-associated kinase
GSK3β: Glycogen synthase kinase 3β
CDK5: Cyclin-dependent kinase 5
CSF: Cerebrospinal fluid
ANOVA: Analysis of variance
PBS: Phosphate-buffered saline
Wt: Wild type
MW: Molecular weight
HRP: Horseradish peroxidase
SSH: Slingshot phosphatase
DMEM/F12: Dulbecco’s Modified Eagle Medium/Nutrient Mixture F-12
PS: Penicillin/Streptomycin
ROI: Region of interest
DIV: Days in vitro
MAP2: Microtubule-associated protein 2
KS: Kolmogorov–Smirnov
CM: Culture medium
