# Supplementary material for: Small molecule modulation of the p75 neurotrophin receptor promotes dendritic spine resilience to pathogenic tau species and reduces their accumulation
Source: Acta Neuropathol Commun. 2026 Mar 5;14:89. doi: 10.1186/s40478-026-02263-5 (PMC13077901; doi:10.1186/s40478-026-02263-5)
Supplement: Supplementary file 1 — Additional file1 (DOCX 573 KB) [file 40478_2026_2263_MOESM1_ESM.docx]

**Supplementary Information:**

**Small molecule modulation of the p75 neurotrophin receptor promotes dendritic spine resilience to pathogenic tau species and reduces their accumulation**

Tao Yang^1^, Yeonglong Ay^1^, Sukhneet Kaur^1^, Robert R. Butler III^1^, Kevin C. Tran^1^, Harry Liu^1^, Vanessa F. Langness^1^, Stephen M. Massa^2,3^, Frank M. Longo^1,4^

^1^Department of Neurology and Neurological Sciences, Stanford University, Stanford, CA 94305

^2^Department of Neurology, San Francisco Veterans Affairs Health Care System

^3^Department of Neurology, University of California, San Francisco, San Francisco, California 94121

^4^Wu Tsai Neuroscience Institute, Stanford University, Stanford, CA 94305

**Supplementary Figures 1-2**

**Description of Supplementary Figures:**

Supplementary Fig. 1. Characterization of S1p fractions from hippocampi of Wt and PS19 mice treated with vehicle or LM11A-31

Supplementary Fig. 2. Uncropped western blots.


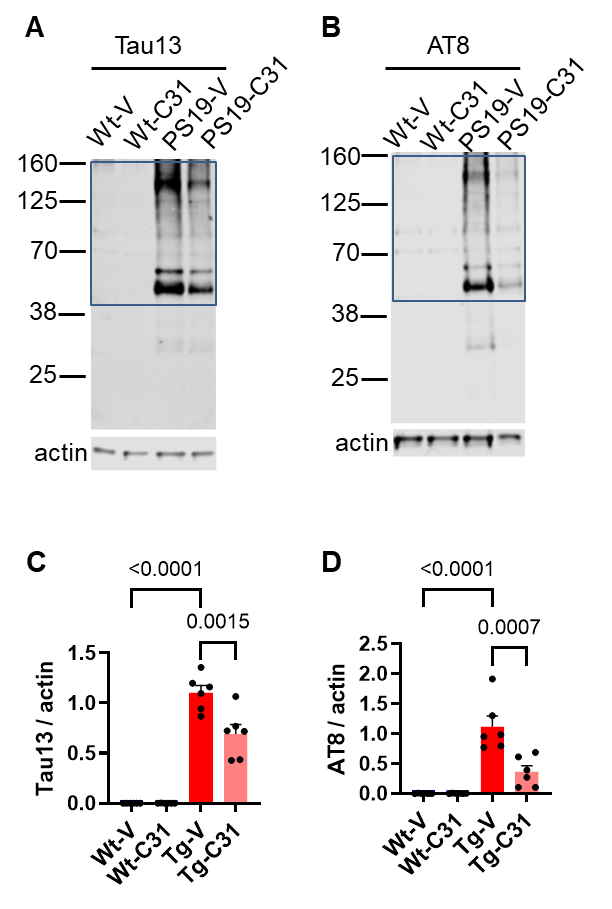


**Supplementary Fig. 1. Characterization of S1p fractions** **from hippocampi of Wt and PS19 mice treated with vehicle or LM11A-31.** Six-month-old Wt and age-matched PS19 mice were treated with vehicle or LM11A-31 for three months. oTau-containing S1p fractions were derived from hippocampal lysates following treatment. (**A**) Western blot analysis using Tau13 demonstrates the presence of oTau (primarily in 100-150 kDa size range) in the S1p fraction of PS19 mice but not of Wt mice. (**B**) Western blot analysis using AT8 antibody further confirms the presence of oTau in the S1p fraction of PS19 mice but not of Wt mice. (**C)** Quantification of the Tau13 blot indicates that LM11A-31 decreases levels of multiple tau species with an overall significant reduction. (**D)** Quantification of the AT8 blot further confirms that LM11A-31 significantly reduces levels of pathological tau. For both **C** and **D**, band intensities for each lane were quantified from the region outlined by the blue box. Statistical significance for both **C** and **D** was assessed using an ordinary one-way ANOVA with Dunnett’s multiple comparisons test (Tau13, F (2, 15) = 63.53, p < 0.0001; AT8, F (2, 15) = 23.51, p < 0.0001). n = 6 mice per condition. Two western blots were run for each mouse sample, with the resulting values averaged to derive one value per mouse.

**
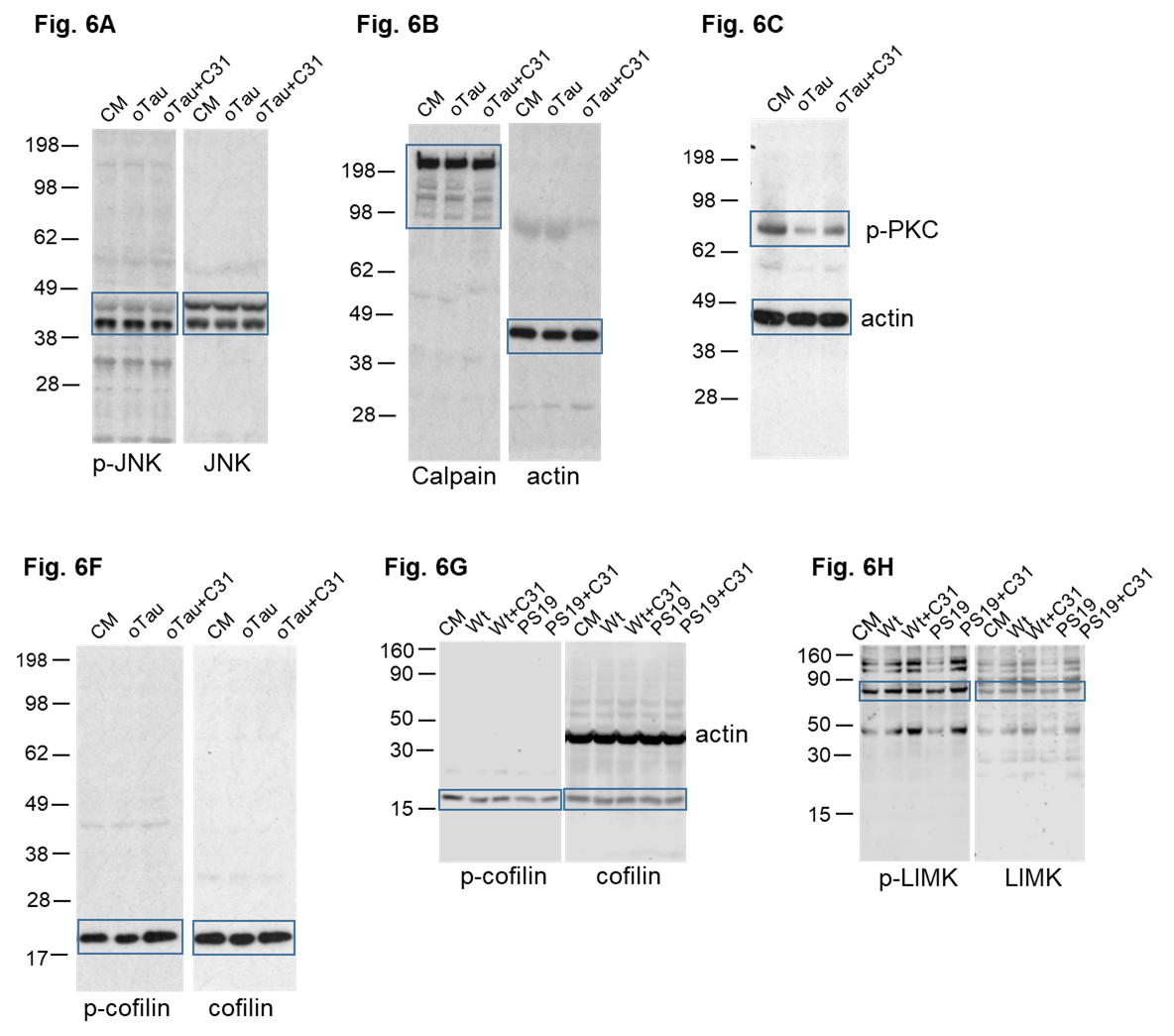
**

**Supplementary Fig. 2. Uncropped western blots.** Each uncropped blot is labeled with the corresponding main figure panel in which the cropped version appears.
